# QTL Mapping and Candidate Gene Analysis for Seed Germination Response to Low Temperature in Rice

**DOI:** 10.3390/ijms23137379

**Published:** 2022-07-02

**Authors:** Nari Kim, Rahmatullah Jan, Jae-Ryoung Park, Saleem Asif, Dan-Dan Zhao, Eun-Gyeong Kim, Yoon-Hee Jang, Gyu-Hyeon Eom, Gang-Seob Lee, Kyung-Min Kim

**Affiliations:** 1 Department of Applied Biosciences, Graduate School, Kyungpook National University, Daegu 41566, Korea; jennynari@hanmail.net (N.K.); rehmatbot@yahoo.com (R.J.); saleemasif10@gmail.com (S.A.); qx288mm@naver.com (D.-D.Z.); dkqkxk632@naver.com (E.-G.K.); uniunnie@naver.com (Y.-H.J.); znfnfn978@naver.com (G.-H.E.); 2 Coastal Agriculture Research Institute, Kyungpook National University, Daegu 41566, Korea; 3 Crop Breeding Division, National Institute of Crop Science, Rural Development Administration, Wanju 55365, Korea; icd92@naver.com; 4 Department of Agricultural Biotechnology, National Institute of Agricultural Sciences, Rural Development Administration, Jeonju 54874, Korea

**Keywords:** low-temperature, seed germination, Cheongcheong Nagdong double haploid, quantitative trait locus, viviparous germination, marker-assisted breeding

## Abstract

Low temperature is a serious threat to the seed emergence of rice, which has become one of the main limiting factors affecting rice production in the world. It is of great significance to find the candidate genes controlling low-temperature tolerance during seed germination and study their functions for breeding new rice cultivars with immense low-temperature tolerance during seed germination. In the current experiment, 120 lines of the Cheongcheong Nagdong Double Haploid (CNDH) population were used for quantitative trait locus (QTL) analysis of low-temperature germinability. The results showed a significant difference in germination under low different temperature (LDT) (15 °C, 20 °C) conditions. In total, four QTLs were detected on chromosome 3, 6, and 8. A total of 41 genes were identified from all the four QTLs, among them, 25 genes were selected by gene function annotation and further screened through quantitative real-time polymerase chain reaction (qRT-PCR). Based on gene function annotation and level of expression under low-temperature, our study suggested the *OsGPq3* gene as a candidate gene controlling viviparous germination, ABA and GA signaling under low-temperature. This study will provide a theoretical basis for marker-assisted breeding and lay the basis for further mining molecular mechanisms of low-temperature germination tolerance in rice.

## 1. Introduction

Seeds are a means of plant survival and propagation; however, changes in the global environment influences their development and germination. Several main biotic and abiotic stress factors badly influence crop yield, especially rice. In addition to that, abiotic stresses such as high/low temperature, drought, salinity, and submergence directly or indirectly influence the physiological status and molecular mechanisms of rice which badly affect yield [1,2,3,4]. The impact of temperature on seed germination is associated with the biosynthesis and signaling of ABA and GA. ABA and GA are the major endogenous regulators that antagonistically control seed dormancy and germination in several plant species [5]. A low temperature during seed development enhances the accumulation of ABA and reduces GA [6,7]. Due to the low temperature, *NCED* (ABA metabolic) and *GA2ox2* (GA catabolic) genes express, which results in decreased ABA and GA level endogenously and directly promotes seed dormancy and reduces seed germination [8,9]. In contrast, it is reported that ABA contents reduces by cold imbibition and the level of ABA biosynthesis and signaling genes (*PYL*, *ABI4*, *ABI5*, *PP2C*) also change with cold imbibition [10]. Seed germination is directly associated with the induction or repression of key genes related to ABA (*NCED3*, *NCED6*, *NCED9*, *CYP707A2* and *ABI5*) and GA (*GA20-oxidase 4* and *GA3-oxidase 1*) biosynthesis and signaling pathways [11]. In addition, the phytohormones such as gibberellin (GA) and abscisic acid (ABA) play essential roles in the regulation of seed dormancy and germination [12,13]. Collectively, temperature regulates seed dormancy through mediating ABA and GA metabolism and signaling.

The ABA biosynthesis and signaling pathways during seed development and germination have been intensively studied until now. In a molecular base study, genes related to ABA biosynthesis, degradation and signaling were identified to play important roles in seed germination and development [14]. Central to ABA signaling in seeds are three core components: PYRABACTIN RESISTANCE/PYRABACTIN-LIKE/REGULATORY COMPONENTS OF ABA RECEPTORS (*PYR/PYL/RCAR*), PROTEIN PHOSPHATASE 2Cs (*PP2Cs*) and SNF1-RELATED PROTEIN KINASE 2s (*SnRK2s*; reviewed in [15]). The identification of *PYL/RCAR* family proteins verified that ABA receptors *PYL/RCAR* are essential ABA signaling components and predominantly function in seeds [16]. A study in rice has revealed that seeds expressing *OsPYL/RCAR* are hypersensitive to ABA during seed germination [17]. *PYLs* release protein phosphatase type 2C (*PP2C*) in the absence of ABA, *PP2C* (another essential component in ABA signaling) acts as a phosphatase [18]. *PP2C* suppresses downstream ABA signaling protein *SnRK2s* due to phosphorylation and inhibits the downstream ABA signaling network [19]. Therefore, *PP2C* acts as a negative regulator in the ABA signaling system [20]. However, in the presence of ABA, *PYR/PYL/RCAR* binds with ABA and *PP2C* to stop the phosphatase activity of *PP2C* which releases and enables the function of the *SnRK2* gene. Our candidate gene *OsGPq3* is also known as *OSK3* and *SnRK*. A recent report shows that *SnRK2* expresses in the nucleus during seed development and germination [21]. *SnRK2* is a positive regulator of downstream genes, which includes *bZIP*, *ABI5*, *ABI4*, and *ABI3* transcription factors, which are the main regulators of ABA-responsive genes [15]. *ABI3* gene expression induces seed dormancy while the *ABI3* mutant induces viviparous seed germination [22]. Viviparous 1 (*VP1*) is an *ABI3* ortholog in maize, shows a critical role in seed dormancy inhibition, promotes viviparous germination and reduces ABA sensitivity [23]. *ABI4* is a key component of the ABA signaling pathway and positively regulates primary seed dormancy by mediating the biosynthesis of ABA and GA. The GA levels of *ABI4* seeds are higher than that of wild rice, suggesting that *ABI4* represses GA biosynthesis [24]. Further study revealed that *ABI4* directly activates key GA catabolic gene *GA2ox7*, whereas GA can repress the expression of key ABA biosynthetic gene *NCED6* in an *ABI4*-dependent manner. In addition, GA can promote *ABI4* degradation while ABA stabilizes it [25]. *ABI5* is a major inducer of seed germination and post-germination growth [26]. Additionally, *SnRK2* plays a key role in the regulation of tiller enhancement. Recently, researchers suggested that the ABA receptor complex (*OsPYL/RCARs*) activates the *SnRK2* protein which phosphorylates tiller enhancer (*APC/CTE*) and interrupts the interaction between tiller enhancer and *OsPYL/RCAR* which subsequently stabilizes the ABA receptor complex (*OsPYL/RCARs*) [27]. In contrast, GA can reduce the level of *SnRK2s* and may promote *APC/CTE*-mediated degradation of *OsPYL/RCARs* [27]. This inference suggested that, GA can reduces ABA signaling by promoting the interaction between tiller enhancer and ABA receptors by reducing *SnRK2* activity which causes subsequent proteasomal degradation of *OsPYL/RCARs* [27].

In this study, a population of 120 lines of Cheongcheong/Nadong Double Haploid (CNDH), derived from two inbred rice lines, (Cheongcheong and Nagdong) was used as a QTL mapping population. The objective of this study was to: (i) analyze seed vigor of this population under LDT conditions; (ii) identify QTL responsible for seed germination under LDT conditions. This study will provide a basis for controlling viviparous germination, ABA and GA signaling during seed dormancy, seed germination and seed development and tiller number regulation.

## 2. Results

### 2.1. Phenotypic Evaluation of Germination Percentage under the LDT Conditions

**Figure 1:**
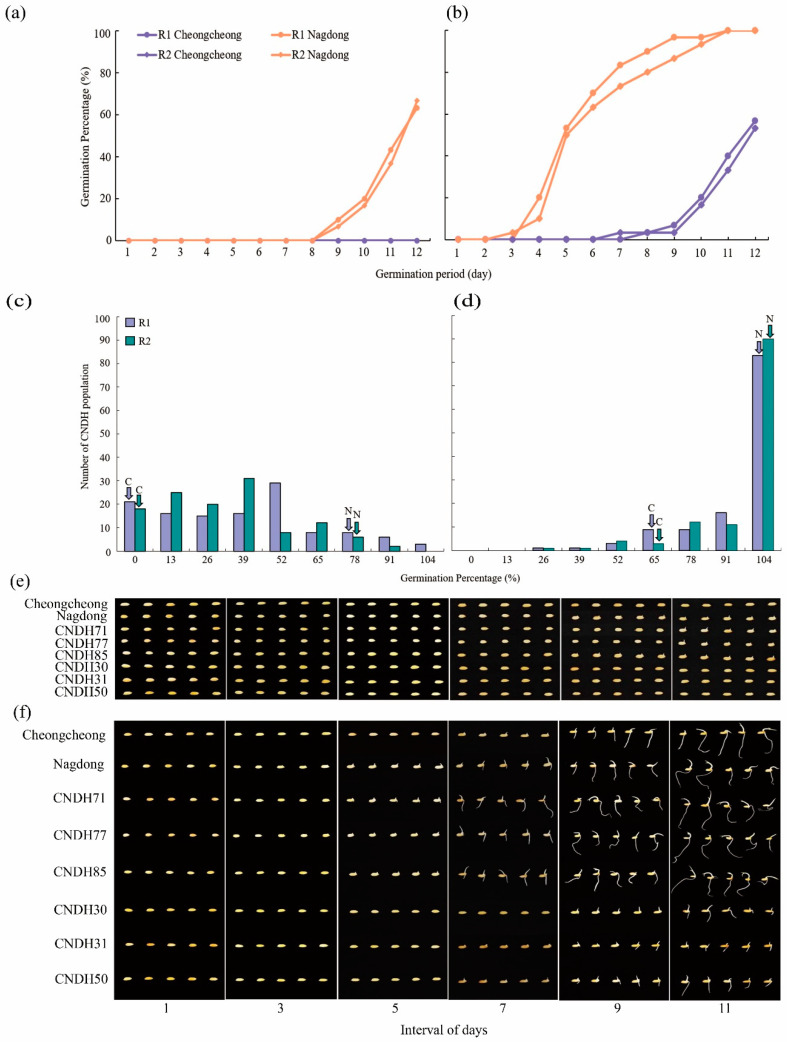
Representation of seed germination and frequency distribution. (**a**,**b**) Represent the GP of Cheongcheong and Nagdong on the daily basis under LDT conditions. (**c**,**d**) Shows frequency distribution for GP under the 15 °C and 20 °C conditions of CNDH population. (**e**,**f**) The seed germination pattern under 15 °C and 20 °C conditions. The highest GP in CNDH population is CNDH71, CNDH77, and CNDH85. The lowest GP in CNDH population is CNDH30, CNDH31, and CNDH50. All pictures were taken every two days after the seeds start germination. C; Cheongcheong, N; Nagdong, R1; replicate 1, and R2; replicate 2.

In the present study, we evaluated the germination percentage (GP) of the CNDH population and their parental lines under LDT conditions as shown in (Figure 1a,b). Under the 15 °C temperature, Cheongcheong were not germinated until the 12th day while the Nagdong started germination after the 9th day and increased GP every day and the data were collected until 12 days. Under the 20 °C temperature, Cheongcheong initiated germination on the 7th day whereas Nagdong started germinating on the 3rd day. Our result shows that the Nagdong shows high germinability at both 15 °C and 20 °C as compared to Cheongcheong. In summary, both parental lines indicated a low GP under the 15 °C conditions compared with the 20 °C conditions and Nagdong showed a higher GP than Cheongcheong among parental lines (Table S1). On the basis of low and high germinability, we selected six lines that are; CNDH71, CNDH77, CNDH85, CNDH30, CNDH31, and CNDH51, from the CNDH population (Figure 1e,f). Among them, CNDH71, CNDH77, and CNDH85 showed 71%, 85%, and 80%, respectively, at 15 °C while 100% germinated at 20 °C. However, CNDH30, CNDH31, and CNDH51 were not germinated at 15 °C and at 20 °C their germination was 28%, 41% and 45%, respectively. The highest germinated lines, CNDH71, CNDH77, and CNDH85 had germinated on the 4th day, 3rd day, and 4th day, respectively, at 20 °C. CNDH30, CNDH31, and CNDH50 which showed low GP started germination on the 8th day, 7th day, and 7th day, respectively, at 20 °C. To statistically validate, our GP results of the CNDH population show normal frequency distribution under both conditions (Figure 1c,d). This experiment was repeated two times.

### 2.2. Analysis of QTLs Associated with Seed Germination

**Figure 2:**
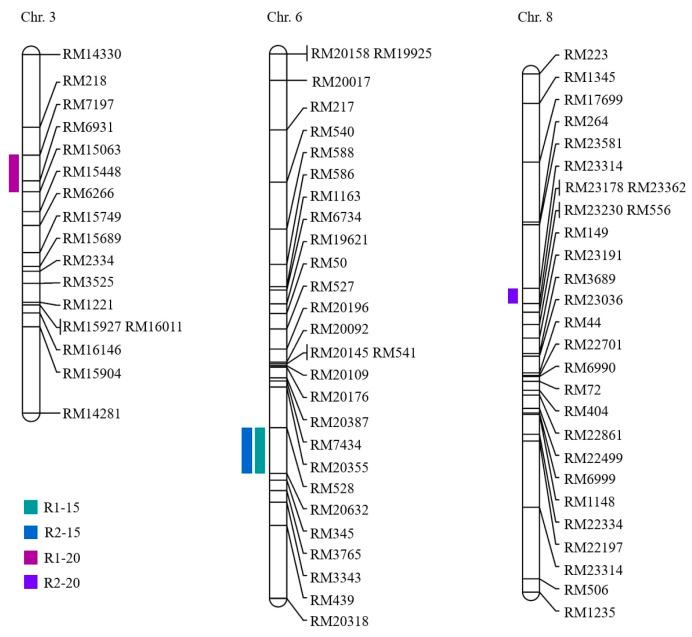
QTL mapping associated with seed germination in the CNDH population. The 4 QTLs were detected in RM7197-RM15063, RM528-RM20632, and RM23314-RM23178 on chromosome 3, 6, and 8, respectively. R1-15; replicate 1 at 15 °C, R2-15; replicate 2 at 15 °C, R1-20; replicate 2 at 20 °C, R2-20; replicate 2 at 20 °C.

Phenotypic data for two repeat-experiment were collected to carry out the QTL mapping. Based on the two repeat-experiment data, four QTLs for seed germination under LDT conditions in the CNDH population were located on chromosomes 3, 6, and 8 (Table 1). The qGP6 and qGP6-1 were detected in RM528-RM20632 on chromosome 6 in both R1 and R2. RM7197-15063 on chromosome 3 was a region related to qGP3. The qGP8 was located at the RM23314-RM23178 on chromosome 8. The qGP3 was identified in RM7197-15063 on chromosome 3 and represented the highest LOD score of 3.36 and the phenotypic variation was 29%. The qGP8 was located on RM23314-RM23178 on chromosome 8 and showed 33% phenotypic variation with a 3.19 LOD score. The qGP6 and qGP6-1 were detected in RM528-RM20632 on chromosome 6 for two repeat-experiment and showed the LOD score of 2.64, and 2.94, respectively. The phenotypic variations were both 30%. Moreover, the alleles of a total of four QTLs associated with seed germination were derived from Cheongcheong. Two QTLs were overlapped on chromosome 6 at marker interval RM528-RM20632 in two repeat-experiments. Finally, the genes related to seed germination under LDT conditions were screened on all chromosomes (3, 6, and 8) (Figure 2).

### 2.3. Searching for Seed Germination Related Genes on the Basis of QTL Mapping

**Figure 3:**
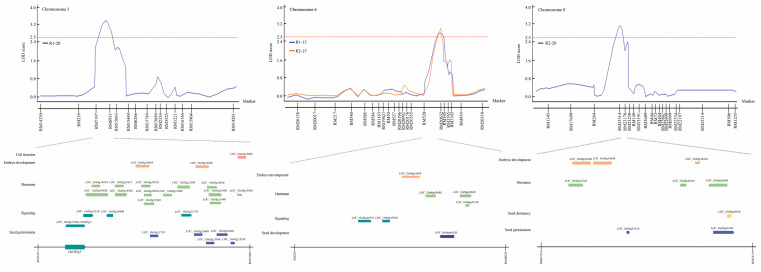
QTL analysis and physical mapping of seed germination-related genes. Genes associated with seed germination corresponding to cell function, embryo development, hormone, signaling, seed development, seed dormancy, and seed germination were identified in RM7197-RM15063, RM528-RM20632, and RM23314-RM23178 on chromosomes 3, 6, and 8, respectively. Among them, *OSK3* (*SnRK*) which plays a key role in the ABA signaling pathway was screened.

As a result of QTL mapping related to seed germination under LDT conditions, a total of four QTLs were detected in RM7197-RM15063, RM528-RM20632, and RM23314-RM23178 on chromosomes 3, 6, and 8, respectively. Depending on the detected four QTLs, genes related to seed germination under LDT conditions were identified using the NCBI database. There were 25, 7, and 9 related genes in RM7197-RM15063, RM528-RM20632, and RM23314-RM23178, respectively, were connected with cell function, embryo development, hormone, signaling, seed development, seed dormancy, and seed germination (Tables S2–S4). Among them, *Os03g0289100*, named *OsGPq3*, which is the *OSK3* (*SnRK*) was selected as the target gene (Figure 3). This gene, *OSK3* (*SnRK*) is involved in the ABA signaling pathway and is a regulator of *ABI3*, *ABI4*, and *ABI5* transcription factor, tiller enhancement, and viviparous germination.

### 2.4. Relative Expression of Genes Related to Seed Germination under LDT

**Figure 4:**
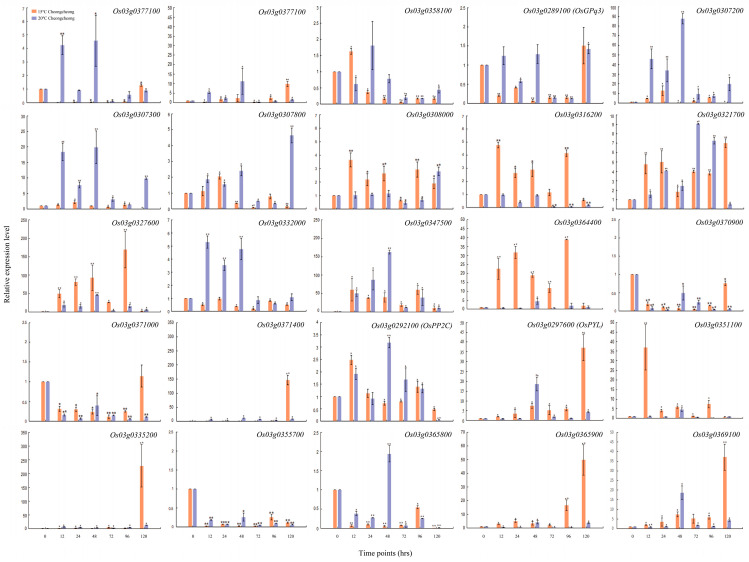
qRT-PCR data about the genes related to seed germination under LDT conditions in Cheongcheong parent. Relative expression levels were analyzed at 0, 12, 24, 48, 72, 96 and 120 h. Each time point is compared with 0 h under 15 °C and 20 °C conditions. Graph bars indicate mean ± standard deviation and asterisks show a significant difference (* *p* < 0.05, ** *p* < 0.01) analyzed by *t*-test.

**Figure 5:**
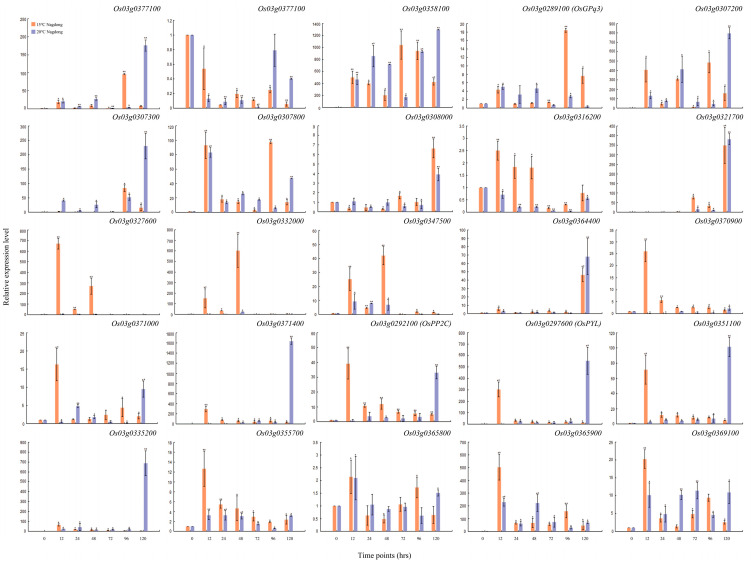
qRT-PCR data about the genes related to seed germination under LDT conditions in Nagdong parent. Relative expression levels were analyzed at 0, 12, 24, 48, 72, 96 and 120 h. Each time point is compared with 0 h under 15 °C and 20 °C conditions. Graph bars indicate mean ± standard deviation and asterisks show a significant difference (* *p* < 0.05, ** *p* < 0.01) analyzed by *t*-test.

In this study, we predicted 25 related genes on different chromosomes and different loci. To narrow down these predicted genes, we further validated them through qRT-PCR (Figure 4 and Figure 5). The results showed that the expression level of *OSK3* (*SnRK*), *PP2C*, and *PYL* was significantly regulated in parental lines under the LDT conditions (Figure 4 and Figure 5). At 15 °C, *Os03g0289100* (*OSK3* or *SnRK*) gene was consistently downregulated until 96 h in Cheongcheong and highly upregulated after 96 h in Nagdong (Figure 4 and Figure 5). On the other hand, at 20 °C it was significantly downregulated at 72 h and 96 h while upregulated after 120 h however, in Nagdong the overall expression was enhanced but, reduced non-significantly after 120 h (Figure 4 and Figure 5). Another candidate *Os03g0292100* (*PP2C*) was significantly regulated by a low temperature (Figure 4 and Figure 5). In Nagdong, the expression was consistently reduced and increased with time at 15 °C and 20 °C, respectively. While in Cheongcheong, after 48 h, the expression of *PP2C* was significantly reduced consistently. The expression level of the third candidate locus (*Os03g297600*, *PYL*) was significantly higher at 15 °C as compared to 20 °C in Cheongcheong except for 48 hrs. While in Nagdong, *PYL* was highly expressed after 12 h and 120 h at 15 °C and 20 °C, respectively.

### 2.5. Analysis of Phylogenetic Tree and Homology Sequence, and Protein Interaction

**Figure 6:**
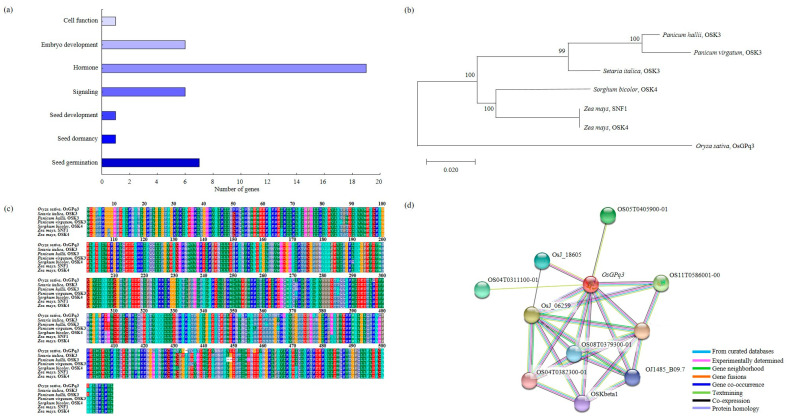
Sequence analysis of *OsGPq3*. (**a**) Related genes of seed germination were involved in cell function, embryo development, hormone, signaling, seed development, seed dormancy, and seed germination. (**b**) To analyze *OsGPq3* and the homology gene, the phylogenetic tree was used. (**c**) The multiple sequence alignment of *OsGPq3*; there is a high similarity among *Oryza sativa*, *Setaria italica*, *Panicum hallii*, *Panicum virgatum*, *Sorghum bicolor*, and *Zea mays*. The accession number of mentioned genes are present in Table S6. (**d**) *OsGPq3* interacts with OS05T0405900-01, OsJ_18605, OS04T0311100-01, OsJ_06259, OS04T0382300-01, OS08T0379300-01, OSKbeta1, OJ1485_B09.7, OsJ_13432, and OS11T0586001-00.

*OsGPq3*, which is an *OSK3* related to the ABA signaling pathway detected in RM7197-RM15063 on chromosome 3. The related genes for seed germination under LDT conditions represented cell function, embryo development, hormone, signaling, seed development, seed dormancy, and seed germination (Figure 6a). The result of phylogenetic tree analysis showed that *OsGPq3* had a genetic similarity among *Panicum hallii*, *Panicum virgatum*, *Setaria italica*, *Sorghum bicolor*, and *Zea mays* which were included in the Gramineae family (Figure 6b). The BLAST analysis using the NCBI database indicated that *OsGPq3* has highly similar sequences to the *OSK3* of *Setaria italica*, *Panicum hallii*, *Panicum virgatum*, *OSK4* of *Sorghum bicolor*, *Zea mays*, and SNF1 of *Zea mays* (Figure 6c). Furthermore, using the *OsGPq3* domain, we identified that *OsGPq3* interacted with 10 proteins (OS05T0405900-01, OsJ_18605, OS04T0311100-01, OsJ_06259, OS04T0382300-01, OS08T0379300-01, OSKbeta1, OJ1485_B09.7, OsJ_13432, and OS11T0586001-00) (Figure 6d). 

## 3. Discussion

Seed germination is the key factor in rice yield. Rice is a sensitive crop at low temperature compared with other main crops. In particular, the low temperature causes harmful influences on the overall process of rice growth [28]. Germination under low-temperature conditions is the main character related to the rice yield and quality and it is essential to verify and grow high-production rice under low-temperature conditions. Below 17 °C, rice is severely affected, mainly resulting in poor germination and seedling establishment, a severe reduction in growth, and lower yield [29]. In short, the germination of seeds is a crucial character to increase the rice yield, which is the main goal of the rice breeding system.

To find related genes, QTL analysis can be used to identify the practical alleles [30]. QTL analysis is an efficient way to identify genes related to germination which control different traits [31]. In the current study, QTL analysis was used for a 120 doubled haploid population from a cross between Cheongcheong (Indica variety) and Nagdong (Japonica variety). QTL analysis of seed germination under low temperature was used to detect target loci that play a vital role in these traits. The results analyzed the seed germination at 15 °C and 20 °C temperatures, which are the quantitative traits that exhibit continuous variation and follow the normal distribution under both the LDT conditions. Based on the two repeated experiment we detected four QTLs related to seed germination under the LDT conditions in the CNDH population, located in RM7197-RM15063, RM528-RM20632, RM23314-RM23178 on chromosome 3, 6, and 8, respectively. The qGP6 and qGP6-1 were detected in RM528-RM20632 on chromosome 6 in both the repeated experiment with LOD scores of 2.64 and 2.94, respectively. RM7197-15063 on chromosome 3 was a region related to qGP3 and showed the highest LOD score of 3.36. The qGP8 was located at the RM23314-RM23178 on chromosome 8 with an LOD score of 3.19. Among them, two QTLs were overlapped on chromosome 6 in the same marker interval RM528-RM20632 in both the repeated experiments. Li et al. and Hua et al. characterized qGP3-1 and qGP-6, respectively, associated with grain number per panicle [32,33]. It is also reported that the GA biosynthesis gene (*OsGA20ox1*) is located in qGP3 for the number of grains per panicle [34]. Consistent with our study, qGP6 was also reported as a major QTL related to seed germination percentage and increases germination by about 3%, and also detected in the RM528-RM340 marker interval [35]. Further reported studies evaluated that qGP6 on chromosome 6 was familiar with the region of qGW-6 for 1000 seed weight and sd6.1 for seed dormancy [36,37]. qGP8 was also previously reported associated with seed germination under osmotic stress detected in the RM6208-RM8264 marker interval [38]. However, the same QTL was detected in linked marker RM7027 associated with the grain number per panicle [39]. These inferences support our study that the four QTLs are linked to seed germination and yield. Among the detected QTLs, 41 germination-related genes were screened, and 25 candidates were identified on chromosome 3. These genes were screened based on gene function annotation. 

To narrow down the most closely related genes, the expression level under the LDT conditions was evaluated through qRT-PCR (Figure 4 and Figure 5). Among the 25 genes detected in the different locus, we selected three candidate genes (*SnRK/OSK*, *PP2C*, *PYL*) according to qRT-PCR validation. Due to previous reports, we assumed that these three genes (*SnRK/OSK*, *PP2C*, *PYL*) are significantly associated with seed germination under low temperature. All three genes are involved in ABA and GA signaling during seed germination under low temperature. ABA plays a key role in several developmental stages such as seed maturation and dormancy [40]. ABA and GA play the most crucial roles as phytohormones in mediating light and temperature-induced transition from seed dormancy establishment to seed germination. ABA promotes dormancy establishment during seed maturation and inhibits seed germination, while GA promotes seed germination [41]. Genetic analysis suggests that *PYR*, *PP2C*, and *SnRK* are important core components of the upstream signal transduction network that regulates the ABA-responsive process, including seed dormancy and seed germination reviewed in [42]. It is mentioned in the Introduction section that ABA physically combines with *PP2C* resulting in the dissociation of *SnRK* from the *SnRK* complex which directly phosphorylates *ABI3*, *ABI4*, and *ABI5* to mediate ABA responses [43]. Our result suggested that *PYR*, *PP2C*, and *SnRK* were significantly upregulated in Nagdong under both low-temperature conditions while Nagdong also showed high GP at both low-temperature conditions. Our results concluded that *PYR*, *PP2C*, and *SnRK* are closely related to seed germination under LDT conditions. These genes are also associated with viviparous germination discussed in the Introduction section. In our study we selected the *SnRK* gene for further future study; however, *PYR* and *PP2C* are also associated with the regulation of seed germination under low temperature.

**Figure 7:**
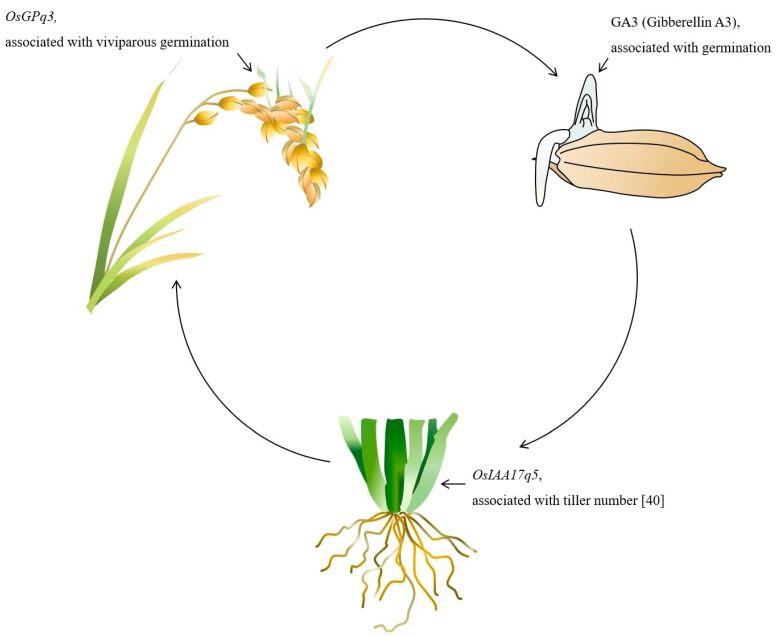
The purposed model of the *OsGPq3* function associated with the viviparous germination, GA regulation, and tiller number regulation.

One of the main functions of the *SnRK* gene is, to regulate ABA and GA mediated via the *ABI4* gene. A recent investigation determined that *ABI4* is significantly involved in GA and ABA antagonistic crosstalk. The *ABI4* enhances ABA biosynthesis and repress GA biosynthesis through the activation of ABA synthesizing and GA repressing genes [44]. This inference assumes that the expression of the *SnRK* gene can discourage viviparous germination via regulation of ABA biosynthesis and GA repression. In another study, it was found that the *SnRK* gene inhibits the tiller enhancer complex (APC/C^TE^) which means that *SnRK* is associated with plant tiller number [27]. On the other hand, recently it was investigated via QTL analysis that the Auxin-related gene (*OsIAA17q5*) is closely related to the plant tiller number [45]. Our proposed model (Figure 7) shows that there is a significant association between the *SnRK*, GA, viviparous germination, and plant tiller pattern. However, molecularly it is poorly understood; therefore, further evaluation is needed to synchronize the crosstalk among the *SnRK*, GA regulation, viviparous germination and plant tiller number. In the next step of our experiment, we will further evaluate our identified gene through overexpression and genome editing with CRISPR-Cas9 technology. Further we will characterize the transgenic rice line by protein expression and ABA and GA signaling under low temperature.

## 4. Materials and Methods

### 4.1. Plant Materials and Preparation of Seeds for Germination Test

A total of 120 CNDH populations were used to analyze seed germination. The CNDH population which originated from another culture of F_1_ hybrid was developed with doubled haploid from a cross between Cheongcheong and Nagdong. The CNDH population was created in 2005 was derived from crossbreeding between Cheongcheong (Indica variety) and Nagdong (Japonica variety). The seeds of the CNDH population were obtained in the experimental field at the Kyungpook National University in Gunwi. The seeds were sterilized with Spotak pesticide (Hankooksamgong, Seoul, South Korea) and put in an incubator at 33 °C for 3 days under dark conditions. The plants were transplanted 30 days after sowing in the experimental field. The planting density of the CNDH population and their parents were 30 × 15 cm. N, P_2_O_5_, and K_2_O were applied at 9.0, 4.5, and 5.7 kg per 10 ha as a fertilizer to prevent diseases and pests (Park et al., 2021). After harvesting, only high-quality seeds were selected for germination.

### 4.2. Evaluation for Seed Germination

The 30 seeds of each CNDH population using three replicates per line were put into the 6 × 9 cm size plastic zipper bag. All plastic zipper bags were put into the beaker and then added distilled water until the whole seeds were immersed. The seeds were placed in an incubator at 20 °C and 15 °C, separately. The germinated seeds were counted every day until 12 days. The emergence of the radicle was considered the initiation of germination. When the radicle length reached approximately 2 mm, the seeds were considered germinated. The GP was calculated as GP (%) = (A total number of germinated seeds/A total number of seeds tested) × 100 [46].

### 4.3. QTL Analysis for GP

The CNDH population genetic map was created by using 778 SSR (simple sequence repeat) markers. Among 778 SSR markers, 423 SSR markers represented polymorphism. Through the PCR amplification, 222 SSR markers were selected and used for QTLs associated with seed germination. To analyze QTLs, Win QTL cartographer 2.5 software was used [47]. Composite interval mapping (CIM) was performed on a whole-genome scan and the LOD score was set at 2.5 [48]. To run this program, the genetic distance between markers, chromosome numbers, genotypic data, target trait values, and marker labels were required [49]. The detected QTLs were named the method proposed by McCouch [50]. 

### 4.4. Gene Information and Statistical Analysis Related to Seed Germination

As for QTL mapping, the identification of candidate genes is a primary factor in the analysis of QTL. To screen the candidate genes and create a physical map, RiceXpro (https://ricexpro.dna.affrc.go.jp/ (accessed on 3 June 2022)) and Rapdb (https://rapdb.dna.affrc.go.jp/ (accessed on 3 June 2022)) were utilized. Open Reading Frames (ORFs) were classified as functions associated with seed germination. Additionally, NCBI (https://www.ncbi.nlm.nih.gov/ (accessed on 3 June 2022)) and BioEdit 7.0 (https://bioedit.software.informer.com/7.0/ (accessed on 3 June 2022)) were used for homologous sequence analysis and STRING for the analysis of protein interactions related to genes. For statistical analysis, the mean and standard deviation were calculated and conducted in three replicates.

### 4.5. Prediction of the Related Genes

On the basis of QTL mapping, the genes related to seed germination under low temperature were screened using Rapdb (https://rapdb.dna.affrc.go.jp/ (accessed on 3 June 2022)) and RiceXpro (https://ricexpro.dna.affrc.go.jp/ (accessed on 3 June 2022)). Rapdb and RiceXpro selected all the ORFs in the target QTL region, and the agriGO tool (http://bioinfo.cau.edu.cn/agriGO/ (accessed on 3 June 2022)) was used to identify the function of all related genes by the gene ontology (GO) enrichment analysis. Based on the classification of gene functions, we searched for genes related to seed germination. For the multiple homologous sequence variation analyses of the related genes comparisons, NCBI (National Center for Biotechnology Information, Bethesda, MA, USA, https://www.ncbi.nlm.nih.gov/ (accessed on 3 June 2022)) and BioEdit 7.0 (https://bioedit.software.informer.com/7.0/ (accessed on 3 June 2022)) were used. Moreover, the MEGA 11 (https://www.megasoftware.net/ (accessed on 3 June 2022)) software was used for the phylogenetic tree. STRING (version 11.0) database (https://string-db.org/ (accessed on 3 June 2022)) was used for protein–protein interaction/association network.

### 4.6. Analysis of Related Gene Expression Level 

To analyze the relative expression level of related genes, seed samples were collected at different times. The total RNA was extracted from seeds of Cheongcheong, Nagdong using the Trizol-based method. The quality and concentration of the total RNA was assessed by ultramicro spectrophotometer ND-2000 (Nanodrop, Wilmington, DE, USA). The RNA was diluted to make a 100 ng/μL concentration and then cDNA was synthesized using the UltraScript 2.0 cDNA Synthesis Kit (PCRBIOSYSTEMS, Wayne, PA, USA). For qRT-PCR, we used StepOnePlusTM RT-PCR System machine (Thermo Fisher Scientific, Seoul, Korea). For the reaction set up, 10 µL 2X Real-time PCR Master Mix (Including SYBR^®^ Green I) (BIOFACT, Daejeon, Korea), 1 µL forward primer (20 pmol/µL), 1 µL reverse primer (20 pmol/µL), 100 ng of cDNA, and the remaining was used as nuclease-free water to make a final volume of 20 µL. The reaction conditions were as follows: polymerase activation at 95 °C for 10 min, denaturation and annealing at 95 °C for 15 s, and extension at 60 °C for 1 min. To calculate the mean and standard deviation, each reaction was performed in three replicates and *OsActin* was used as a housekeeping gene. The genes primer list is shown in (Table S5).

## 5. Conclusions

In summary, we carried out QTL analysis to identify the seed germination-related genes under LDT conditions. The results showed that four QTLs were detected in RM7197-RM15063, RM528-RM20632, and RM23314-RM23178 on chromosomes 3, 6, and 8, respectively. A total of 41 genes were screened on all chromosomes, among them, 25 related genes were selected by gene function annotation and further identified through qRT-PCR. Therefore, our study suggests that *OsGPq3* (*OSK*/*SnRK*) gene involved in viviparous germination, ABA signaling pathway, and tiller enhancement will provide a basis for further study associated with low-temperature germinability in rice.

## Figures and Tables

**Table 1:** QTL associated with the germination percentage in the CNDH population.

Replicates	QTLs	Chr.	Marker Interval ^z^	LOD	Additive Effect ^y^	R^2 x^	Increasing Effect ^w^
1	qGP6	6	RM528-RM20632	2.64	7.94	0.3	Cheongcheong
	qGP3	3	RM7197-RM15063	3.36	6.62	0.29	Cheongcheong
2	qGP6-1	6	RM528-RM20632	2.94	7.16	0.3	Cheongcheong
	qGP8	8	RM23314-RM23178	3.19	5.37	0.33	Cheongcheong

^z^ The markers which are in the significance threshold. ^y^ The positive values indicate the contribution from the mother plant. ^x^ The phenotypic variation. ^w^ The source of the allele generating an increase in assessed traits.

## Data Availability

The data presented in this study are available on request from the corresponding author.

## Supplementary Materials

The following supporting information can be downloaded at: https://www.mdpi.com/article/10.3390/ijms23137379/s1.
