# Supplementary material for: QTL Mapping and Candidate Gene Analysis for Seed Germination Response to Low Temperature in Rice
Source: Int J Mol Sci. 2022 Jul 2;23(13):7379. doi: 10.3390/ijms23137379 (PMC9266303; doi:10.3390/ijms23137379)
Supplement: Supplementary file 1 [file ijms-23-07379-s001.zip › ijms-1787210-supplementary.pdf]

**Supplementary tables**

**Table S1.** Phenotypic values of germination percentage in CNDH population

| Replicates | Temperature (℃) | Parents | | CNDH Population | | |
|---|---|---|---|---|---|---|
| | | Cheongcheong | Nagdong | Max. | Min. | Mean |
| 1 | 15 | 0.0 ± 0.0 | 70.0 ± 3.3 | 100.0 | 0.0 | 38.0 ± 31.9 |
| 2 | | 0.0 ± 0.0 | 66.7 ± 1.7 | 100.0 | 0.0 | 37.6 ± 31.3 |
| 1 | 20 | 56.7 ± 1.7 | 100.0 ± 0.0 | 100.0 | 23.3 | 88.2 ± 17.6 |
| 2 | | 55.0 ± 0.8 | 100.0 ± 0.0 | 100.0 | 25.0 | 88.9 ± 16.6 |

The data are presented as mean ± standard deviation. The GP was investigated under different temperature conditions.

**Table S2.** 25 genes related to seed germination were screened from the interval RM7197-RM15063 on chromosome 3.

| Function | Locus | Description | No. of Genes |
|---|---|---|---|
| Cell function | LOC_Os03g25990 | Similar to Expansin (Expansin2) | 1 |
| Embryo development | LOC_Os03g20910 | Homeodomain-like containing protein | 2 |
| | LOC_Os03g24380 | Similar to Glutathione peroxidas | |
| Hormone | LOC_Os03g19420 | Nicotianamine synthase 2 (EC 2.5.1.43) (S-adenosyl-L-methionine:S- adenosyl-L-methionine:S-adenosyl-methionine 3-amino-3- carboxypropyl-transferase 2) (*OsNAS2*) | 13 |
| | LOC_Os03g19427 | Nicotianamine synthase 1 (EC 2.5.1.43) (S-adenosyl-L-methionine:S- adenosyl-L-methionine:S-adenosyl-methionine 3-amino-3- carboxypropyl-transferase 1) (*OsNAS1*) | |
| | LOC_Os03g19480 | SET domain-containing protein | |
| | LOC_Os03g19500 | Ubiquitin-conjugating enzyme, E2 domain containing protein | |
| | LOC_Os03g20120 | Similar to Galactinol synthase (Fragment) | |
| | LOC_Os03g20550 | Similar to WRKY transcription factor 55 | |
| | LOC_Os03g21040 | Ricin B-related lectin domain containing protein | |
| | LOC_Os03g21400 | Cytochrome P450 family protein | |
| | LOC_Os03g22590 | Similar to Senescence-associated protein (SAG29) | |
| | LOC_Os03g24930 | Similar to Phytosulfokine receptor-like protein | |
| | LOC_Os03g25480 | Similar to Cytochrome P450 | |
| | LOC_Os03g25490 | Similar to Cytochrome P450 | |
| | LOC_Os03g25500 | Cytochrome P450 family protein | |
| Signaling | LOC_Os03g17980 | *OSK3* (*OSK5*) | 4 |
| | LOC_Os03g18150 | Protein phosphatase 2C family protein | |
| | LOC_Os03g18600 | Streptomyces cyclase/dehydrase family protein | |
| | LOC_Os03g22770 | Similar to CONSTANS-like protein CO9 (Fragment) | |

| | | | |
|---|---|---|---|
| Seed germination | LOC_Os03g21710 | Similar to WRKY1 (WRKY transcription factor 17) | 5 |
| | LOC_Os03g23960 | IQ calmodulin-binding region domain containing protein | |
| | LOC_Os03g25030 | Lipolytic enzyme, G-D-S-L family protein | |
| | LOC_Os03g25040 | Lipolytic enzyme, G-D-S-L family protein | |
| | LOC_Os03g25350 | Plant lipid transfer/seed storage/trypsin-alpha amylase inhibitor domain containing protein | |

**Table S3.** 7 genes related to seed germination were screened from the interval RM528-RM20632 on chromosome 6.

| Function | Locus | Description | No. of Genes |
|---|---|---|---|
| Embryo development | LOC_Os06g45640 | Similar to Nuclear Y/CCAAT-box binding factor C subunit NF-YC | 1 |
| Hormone | LOC_Os06g45960 | Cytochrome P450 family protein | 3 |
| | LOC_Os06g46680 | Cytochrome P450 family protein | |
| | LOC_Os06g46740 | Cupredoxin domain containing protein | |
| Signaling | LOC_Os06g44970 | Similar to Auxin efflux carrier protein | 2 |
| | LOC_Os06g45300 | Serine/threonine protein kinase-like protein | |
| Seed development | LOC_Os06g46330 | Protein kinase-like domain containing protein | 1 |

**Table S4.** 9 genes related to seed germination were screened from the interval RM23314-RM23178 on chromosome 8.

| Function | Locus | Description | No. of Genes |
|---|---|---|---|
| Embryo development | LOC_Os08g34380 | Similar to SERK1 (Fragment) | 3 |
| | LOC_Os08g34640 | Similar to Receptor-like protein kinase precursor (EC 2.7.1.37). Splice iso-form INRPK1a | |
| | LOC_Os08g36440 | TB2/DP1 and HVA22 related protein family protein | |
| Hormone | LOC_Os08g34210 | Glyceraldehyde-3-phosphate dehydrogenase | 3 |
| | LOC_Os08g36310 | Cytochrome P450 family protein | |
| | LOC_Os08g36860 | Cytochrome P450 family protein | |
| Seed dormancy | LOC_Os08g36910 | Alpha-amylase isozyme 3D precursor (EC 3.2.1.1) (1,4-alpha-D-glucan glucanohydrolase) | 1 |
| Seed germination | LOC_Os08g35110 | Auxin responsive SAUR protein family protein | 2 |
| | LOC_Os08g36790 | TRAB1 (BZIP transcription factor) | |

**Table S5.** List of primers used for qRT-PCR experiments.

| Name | Forward primers (5'-3') | Reverse primers (5'-3') |
|---|---|---|
| *Os03g0377100* | GCAAGTCCATCGTGGTGAC | AGTAGGAGTGCCCGTTGATG |
| *Os03g0325600* | ATTACCTGCAGCGACGATCA | TCGGAGTAGAGAGAGAGCGAT |
| *Os03g0358100* | GGGAAGGTGCTAATCGTCGT | CGCAAGAACTGATTGCAGGG |
| *Os03g0289100* (*OSK3*) | GCATGGTTTCTGTGACACACC | TGACGCAAGATCCAAGCTGT |
| *Os03g0307200* | GTGTTCGACAACTACGACCG | CAGACGGATAGCCTCTTGG |
| *Os03g0307300* | CGTACTACGGCAACTACGTCA | AGACGGACAGCTCCTTGTTG |
| *Os03g0307800* | CCTCCGATTCCTCTTCCCAAC | CCCATGGAGACGGCAATCAA |
| *Os03g0308000* | GATGAGCATGCACACAAGGC | AGTCCAAATGCAGCCAAGGT |
| *Os03g0316200* | GTACAAGCCGATCCCACTGA | AGCTGGACGTTCTCTGGATG |
| *Os03g0321700* | ACGGCATTCGGAAGCCTATT | AGATTGGAGCTCAGTGCAGG |
| *Os03g0327600* | CACCTTCAAGATCTTCTGCCG | TCGAAGTTGAGGCGGATGTT |
| *Os03g0332000* | TGGTCAGGGCTTTGCTATGG | CATCTTCCCTTGCACTCTCGT |
| *Os03g0347500* | ATTGGTCTTTGCTGTGGGGA | CCGATAAAACGTCGGCACTG |
| *Os03g0364400* | TCGTCTACGAGTTCATGCCG | TCCGAGTCGAGGAGGATGTT |
| *Os03g0370900* | CAATGATCGGCAAAGGGCTG | GTTGCTATGGCAATGGCCTG |
| *Os03g0371000* | AGGCTCTATGGTCCTGCTCT | CTCCTTGTCCCGGTGCATAA |
| *Os03g0371400* | CGTGGACCTGCCTACAAGTT | AGGTGCTATCCTTGGGAGGT |
| *Os03g0292100* (*OsPP2C*) | GACAGTGGCGAGACTGAACA | TTAGACACAACGAGGCCACC |
| *Os03g0297600* (*OsPYL*) | AGCACCGCCTCAAGAACTAC | GTCTTGGCGAGAGACTGGAG |

| | | |
|---|---|---|
| *Os03g0351100* | CCATCTTCCCCCAAGATTCAGA | TAGAACTTGCATTGGCGTGC |
| *Os03g0335200* | CCGCAACACTAGAAGACGGT | TGTGCGTGCACCTGTAGTAG |
| *Os03g0355700* | GGAGAGGAAGAGGAGGGGAT | TTCACCTTCTTGTCCGGCTT |
| *Os03g0365800* | CGACTTCCTAGCGGATGATCT | GCCTCCAACCTCACCCATTA |
| *Os03g0365900* | AGAGCGCAAGGACATTACG | GAGAAACCTCGGAATGCAGC |
| *Os03g0369100* | TGCAAGAAGTACGAGGGGTG | AGACGACCTTCTCCATGCAC |

**Table S6.** Accession number of genes mentioned in figure 6C.

| Specie name | Gene name | Accession number |
|---|---|---|
| *Oryza sativa* | *OsGPq3* | NM_001402138<br>XM_015776763 |
| *Seteria italica* | *OSK3* | XM_012843081 |
| *Panicum hallii* | *OSK3* | XM_025940294 |
| *Panicum virgatum* | *OSK3* | XM_039930633 |
| *Sorghum bicolor* | *OSK4* | XM_021450779 |
| *Zea mays* | *SNF1* | NM_001301590<br>XM_008671745 |
| *Zea mays* | *OSK4* | PWZ55924 |
